# *Plasmodium falciparum*-Infected Erythrocytes and IL-12/IL-18 Induce Diverse Transcriptomes in Human NK Cells: IFN-α/β Pathway *versus* TREM Signaling

**DOI:** 10.1371/journal.pone.0024963

**Published:** 2011-09-16

**Authors:** Elisandra Grangeiro de Carvalho, Michael Bonin, Peter G. Kremsner, Jürgen F. J. Kun

**Affiliations:** 1 Institute for Tropical Medicine, University of Tübingen, Tübingen, Germany; 2 Department of Medical Genetics, Institute of Human Genetics, University of Tübingen, Tübingen, Germany; Ludwig-Maximilians-Universität München, Germany

## Abstract

The protective immunity of natural killer (NK) cells against malarial infections is thought to be due to early production of type II interferon (IFN) and possibly direct NK cell cytotoxicity. To better understand this mechanism, a microarray analysis was conducted on NK cells from healthy donors PBMCs that were co-cultured with *P. falciparum* 3D7-infected erythrocytes. A very similar pattern of gene expression was observed among all donors for each treatment in three replicas. Parasites particularly modulated genes involved in IFN-α/β signaling as well as molecules involved in the activation of interferon regulatory factors, pathways known to play a role in the antimicrobial immune response. This pattern of transcription was entirely different from that shown by NK cells treated with IL-12 and IL-18, in which IFN-γ- and TREM-1-related genes were over-expressed. These results suggest that *P. falciparum* parasites and the cytokines IL-12 and IL-18 have diverse imprints on the transcriptome of human primary NK cells. IFN-α-related genes are the prominent molecules induced by parasites on NK cells and arise as candidate biomarkers that merit to be further investigated as potential new tools in malaria control.

## Introduction

Infections caused by malaria parasites, especially by the species *Plasmodium falciparum*, remain a serious world health concern. The innate and adaptive arms of the immune system are involved in immunity to malaria, however, the engaged macrophages, dendritic cells, γδ T cells, natural killer (NK) cells, and NKT cells fail to fully eliminate the infection [1], [2].

Characterized by cytotoxicity and cytokine secretion, NK cells play a critical role as the front line of defence against pathogens and tumor cells. Within the setting of malaria, several studies have elucidated the interactions between NK cells, infected erythrocytes (iRBCs) and other immune cells leading to specific NK responses to *P. falciparum* [3]–[5]. Experiments performed with NK cells derived from malaria-naive or infected individuals showed that these cells have cytolytic activity against *P. falciparum*-iRBCs that is possibly mediated by FAS and Granzyme B [6], [7]. The DBL-alpha domain of *P. falciparum*-infected erythrocyte membrane protein 1 (PFEMP1) was identified as the molecule through which NK cells recognize iRBCs [8]. MYD88-associated IL18R in NK cells was shown to be the major indirect sensor for *P. falciparum* infection [9].

Experimental evidence suggested that, in addition to their up-regulation of CD69 and CD25 after contact with iRBCs, NK are one of the first cells to produce IFN-γ in response to *P. falciparum* infection [3], [5]. This event was described to be dependent on cross-talk with accessory cells either via direct or indirect interactions. The possible bidirectional interplay between ICAM and LFA-1 on NK cells and macrophages was shown to be important for NK cell up-regulation of CD69 and IFN-γ secretion [10]. Indirectly, the production of cytokines by accessory cells, especially IL-12, IL-18, IFN-alpha and IL-2, was shown to boost NK cell activation and IFN-γ release in response to iRBCs [11].

However, the magnitude of IFN-γ release by NK cells is known to be heterogeneous among individuals, possibly influencing susceptibility to disease [5]. In this line, qualitative and quantitative differences in NK subsets found in malaria patients were linked to the severity of the disease [3]. In addition, correlations between KIR genotype and NK cell responsiveness to iRBCs have been reported [4].

Microarray techniques have been widely used for research as well as for diagnostic purposes. Therefore, applications pertinent to host-microorganism interactions may be a good predictor of the biological processes thereby involved. In this study, Affymetrix oligonucleotide microarrays were used to examine the gene expression profile of primary NK cells from three healthy donors that were co-cultured with *P. falciparum* parasites. This pattern of gene expression was compared to the same NK cells following stimulation with IL-12+IL-18.

The response of NK cells to malaria has been the topic of several studies over the previous few years, but there is still a lack of information regarding the impact of *P. falciparum* on NK cells at a transcriptional level. A greater understanding of the NK cell mechanisms of sensing and responding to iRBCs is needed seeking the advantages of NK cell-targeted vaccines development against malaria.

## Materials and Methods

### Ethics statement

The three healthy individuals who served as NK cell donors are themselves authors of this study. Therefore, acquisition of verbal informed consent was considered sufficient by the ethics committee for the study approval. Verbal consent was obtained in the presence of a witness unrelated to the study, who has attested to its voluntary character in a signed document. The study was approved by the Ethics Committee of the University of Tübingen, Germany.

### *P. falciparum* culture

The *P. falciparum* laboratory strain 3D7 was maintained in continuous culture as described elsewhere [12] and frequently tested for mycoplasma contamination by PCR prior to co-cultivation with NK cells. Parasites were constantly synchronized with 5% sorbitol. Mature schizont-iRBCs were harvested by magnetic cell sorting with LD columns (MACS; Miltenyi Biotec, Berg. Gladbach, Germany). Schizonts' purity (>90%) and red blood cell integrity were confirmed by Giemsa stain.

### PBMCs preparation

Venous blood was collected and immediately processed. Three healthy adults (donors E, K and V) with no prior exposure to *Plasmodium* parasites were used in this study. Samples were collected into 9 ml ammonium heparin tubes (16I.U. heparin/ml blood; S. Monovette) and diluted 1∶1 with RPMI 1640 (Sigma Aldrich). Peripheral blood mononuclear cells (PBMCs) were isolated by density gradient centrifugation with Ficoll Paque TM plus (GE Healthcare). The cells were washed twice with 2% FBS in RPMI 1640; resuspended in culture medium (RPMI 1640) containing 5% autologous serum, 1% 100× PenStrep (Invitrogen) and 2 mM L-Glutamine (Invitrogen); transferred to 24-well flat-bottomed plates (Nunc); and cultured as described below.

### PBMC/parasite co-incubation

Freshly isolated mononuclear cells from donors E and K were incubated under four different conditions: in culture medium alone (CM or untreated); with a mixture of IL-12 and IL-18 (Peprotech and MBL, respectively; 200 ng/10^6^ cells each); or with schizont-iRBCs or with uninfected erythrocytes (uRBCs) at a ratio of three RBCs for each mononuclear cell. PBMCs from donor V were incubated under two different conditions: with iRBCs and with culture medium. Co-cultures were maintained at 37°C and 5% CO2 for 24 hours. After the incubation, NK cells were isolated from PBMCs, checked for purity by FACS, and subjected to RNA extraction as described below. The experiment was repeated three times (1–3 weeks apart) for each one of the three donors.

To evaluate the activation pattern of each donor's NK cells, PBMCs were likewise incubated with schizont-iRBCs (1 PBMC : 3 iRBC), with iRBCs together with human IFN-alpha2b (Myltenyi Biotec; 500 U/10^6^ cells), or with a mixture of IL-12 and IL-18 (200 ng/10^6^ cells each) and also kept in culture medium alone. After 24 hours at 37°C and 5% CO_2_, cells were harvested, iRBCs were lysed and PBMCs were stained for flow cytometry.

### Cell surface and intracellular staining for flow cytometry

The following antibodies were used for flow cytometric staining: CD56-FITC, CD3-PE, CD3-APC, CD69-PE, intracellular IFN-γ-PE, 7AAD and the appropriate isotype controls (all from BD biosciences). Extracellular staining of cells was performed according to the manufacturer's instructions. For intracellular staining of IFN-γ, Brefeldin A solution (Biolegend) was added 4 hours before the end of the incubation period and cells were fixed and permeabilized with Cytofix/Cytoperm Fixation/Permeabilization Kit (BD Biosciences) according to the manual instructions.

### Isolation of NK cells

After 24 hours of co-incubation, cells were harvested, separately treated with BD Pharm Lyse lysing buffer (BD) for RBC rupture, and washed twice with auto-MACS Rinsing Solution (Miltenyi Biotec). NK cells were enriched from PBMCs by negative selection with the NK Cell isolation Kit (Miltenyi Biotec) according to the manufacturer's instructions. NK cells were counted and tested for viability with trypan blue, and purity was determined by flow cytometry. A purity of ≥93% CD56^+^CD3^−^ cells was considered acceptable (Figure S1).

### RNA extraction and Microarrays

Total cellular RNA was isolated from the enriched NK cells with RNeasy Mini Kit (Qiagen, Hilden, Germany). The quality of each specimen was checked using an Agilent BioAnalyzer 2100 (Agilent, Germany). RNA was processed for Affymetrix Gene Chips using the Affymetrix Whole Transcript Sense Target Labeling Kit (Affymetrix, Santa Clara, USA). Fragmented and labeled cDNA were hybridized onto human HuGene1.0 ST Gene Chips (Affymetrix). The staining of biotinylated cDNA and scanning of arrays were performed according to the manufacturer's recommendations. The complete microarray data is deposited at the Gene Expression Omnibus (GEO) of the National Center for Biotechnology Information (NCBI) under the series number GSE24791. Validation of the method was performed by RT-PCR.

### Rea- time PCR

cDNA was synthesized from total RNA using the Quantitec Reverse Transcription kit (Qiagen) with the elimination of Genomic DNA according to the manufacturer's instructions. Amplification of IFIT1, IFIT3 and IFI44L genes was carried out in duplicates using the Rotor Gene Syber Green PCR Kit (Qiagen) with Quantitec Primer Assay (both from Qiagen). Cycling conditions for fast two-step RT-PCR on Rotor-Gene cycles were applied according to the Primer Assay Handbook (Qiagen). Levels of target mRNA expression were determined using the 2^−ΔΔCT^ method with GAPDH as the endogenous reference gene and the CM samples as calibrators.

### NK cells expansion and co-culture with parasites for growth inhibition assay

PBMCs and NK cells from donor E were respectively purified and isolated as described above. NK cells (≥93% CD56^+^CD3^−^) were cultured in IMDM medium (Sigma) with 5% autologous serum, 200 U/ml IL-2 (Peprotech) and irradiated JY cells at a 1∶3–3∶1 ratio (NK∶JY). Purified NK cells expanded for 2–4 weeks (eNK) were co-incubated at 37°C with ring stage 3D7-iRBCs in parasite growth medium at a 1∶3 or 5∶1 ratio (NK∶3D7) in parasite atmosphere. Additionally, IFN-alpha2b (500 U/10^6^ cells) or a mixture of IL-12 and IL-18 (200 ng/10^6^ cells each) were added to the system. Parasites were cultured alone as a control and the initial parasitemia was set as 0,05% in 1,5% hematocrit. After 24 h and 48 h of incubation, culture samples were frozen at −20°C, then thawed and inhibition of parasite growth was quantified by a Histidine-Rich Protein 2 (HRP2) ELISA assay performed as described elsewhere [13].

### Bioinformatic analysis

Raw CEL-files were imported into Expression Console 1.0 (Affymetrix, Santa Clara). RMA16 was used for array normalization and signal calculation. Normalized signal values were imported into Genespring 11 (Agilent Technologies). Significance was calculated using a t-test without multiple testing correction, selecting all the transcripts with a minimum change in expression level of 1.5-fold together with a p-value of <0.05. Subsequently, transcripts in common for all donors in each treatment were compiled and gene network analysis and functional categorization was performed with Ingenuity Pathway Analysis (IPA) (www.ingenuity.com). The p-value associated with a biological process or pathway annotation for IPA is a measure of its statistical significance with respect to the Functions/Pathways/Lists Eligible molecules for the dataset and a reference set of molecules (which define the molecules that could possibly have been Functions/Pathways/Lists Eligible). The p-value is calculated with the right-tailed Fisher's Exact Test. The ratio is calculated by taking the number of genes from the dataset that participate in a Canonical Pathway, and dividing it by the total number of genes in that Canonical Pathway. The ratio indicates the percentage of genes in a pathway that were also found in the uploaded gene list (or the Functions/Pathways/Lists Eligible genes if a cut off was specified) and is therefore useful for determining which pathways overlap with most of the genes in the dataset.

## Results

### *P. falciparum-*iRBCs induce the up-regulation of type I interferon-related genes in NK cells

**Figure 1:**
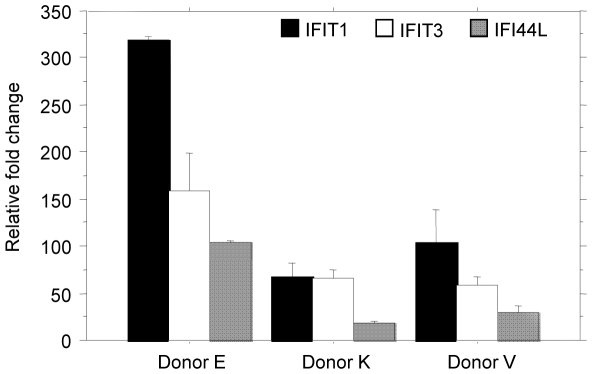
Validation of the microarray results by RT-PCR. Three representative iRBCs-induced NK genes are depicted. Values represent the mean of the relative fold change obtained for each replicate per donor. Levels of target mRNA expression were determined using the 2^−ΔΔCT^ method with GAPDH as the endogenous reference gene and the untreated samples (CM) as calibrators.

Affymetrix microarrays were used to evaluate the gene expression profile of NK cells isolated from PBMCs (purity ≥93%; Figure S1) that were incubated with iRBCs to detect the changes that *Plasmodium*-iRBCs impose on the gene repertoire of NK cells. The analysis showed that 192 genes were commonly modulated for all donors in response to iRBCs contact. Of those genes, nine were down-regulated and 183 were up-regulated in comparison to untreated cells (Table S1). The expression profile was characterized by the induction/suppression of genes mainly related to immune response and response to virus (*IFIT1*, *IFIT3*, *OAS3*, *KLRG1*), chemotaxis (*CXCL10*, *CCR1*, *CCL4L1*), signal transduction (*CD38*, *IFITM1*, *FAS*), regulation of transcription (*STAT2*, *IRF7 STAT3*), intracellular signaling pathway (*JAK*, *RASGRP3*, *RASGRP2*), and NK cytotoxicity (*SLAMF7*), among others. A summary of the most highly up- and down-regulated genes is depicted in Table 1. A portion of the most highly up-regulated genes (fold change ≥10) encode proteins mostly related to interferon signaling (*IFIT1*, *IFIT3*, *IFI44L*, *IFIT2*, *IFI6*, *and IFI44*), especially via IFN-α. The most highly down-regulated genes (fold change ≤−1.5) are mainly involved in chromatin assembly, receptor activity in the immune response and signal transduction. Three representative genes were chosen for microarray validation by RT-PCR (Figure 1).

**Table 1:** Top up/down-regulated genes on NK cells due to co-culture with *P.falciparum*-iRBCs.

Symbol	Aff. ID	FC	Location	Type
IFIT1	7929065	34,50	Cytoplasm	other
RSAD2	8040080	21,55	unknown	enzyme
IFIT3	7929052	19,71	Cytoplasm	other
OAS3	7958895	15,48	Cytoplasm	enzyme
MX1	8068713	15,42	Nucleus	enzyme
IFI44L	7902541	15,33	unknown	other
IFIT2	7929047	12,47	unknown	other
IFI6	7914127	12,10	Cytoplasm	other
OAS1	7958884	11,60	Cytoplasm	enzyme
IFI44	7902553	11,32	Cytoplasm	other
MX2	8068697	11,00	Nucleus	enzyme
CBX7	8076185	−1,79	Nucleus	other
KLRG1	7953835	−1,83	Plasma MB	other
RASGRP2	7949104	−1,91	Cytoplasm	other
SYNE1	8130211	−1,92	Nucleus	other
HERC1	7989516	−1,96	Cytoplasm	other
CMKLR1	7966089	−2,03	Plasma MB	GPCR
AHNAK	7948667	−2,27	Nucleus	other
FGR	7914112	−2,49	Nucleus	kinase
PTGDR	7974363	−2,58	Plasma MB	GPCR

iRBCs-infected erythrocytes; Aff. ID-affymetrix identification; FC-fold change; MB-membrane; GPCR-G protein coupled receptor.

### Top Networks and Pathways related to iRBCs-induced genes

**Figure 2:**
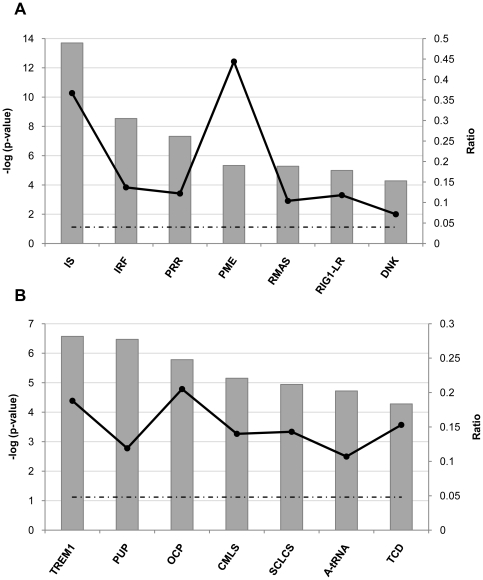
Top canonical pathways associated with 3D7- and IL-12/IL-18-induced NK cell genes. Canonical pathways were obtained using the Ingenuity System upon analysis of the genes differentially modified in NK cells. Top canonical pathways are indicated by the grey bars on the x-axis. The association of the data set with the given pathway is depicted in the y-axes and was determined based on the p-value and on the ratio (strength of the association - black line). The threshold (dashed black line) is shown at p<0.05. **A. 3D7-induced**: IS: Interferon Signaling; IRF: Activation of Interferon Regulatory Factor family of transcription factors by Cytosolic Pattern Recognition Receptors; PRR: Role of Pattern Recognition Receptors in Recognition of Bacteria and Viruses; PMS: Pathogenesis of Multiple Sclerosis; RMAS: Retinoic acid Mediated Apoptosis Signaling; RIG1-LR: Role of Retinoic Acid Inducible gene 1-like receptors in Antiviral Innate Immunity; DNK: Crosstalk between Dendritic Cells and Natural Killer Cells. **B. IL-12+IL-18-induced**: TREM1: Triggering Receptor Expressed in Myeloid Cell 1 Signaling; PUP: Protein Ubiquitination Pathway; OCP: One carbon Pool by Folate; CMLS: Chronic Myeloid Leukemia Signaling; SCLCS: Small Cell Lung Cancer Signaling; A-tRNA: Aminoacyl-tRNA Biosynthesis; TCD: T Helper Cell Differentiation.

**Figure 3:**
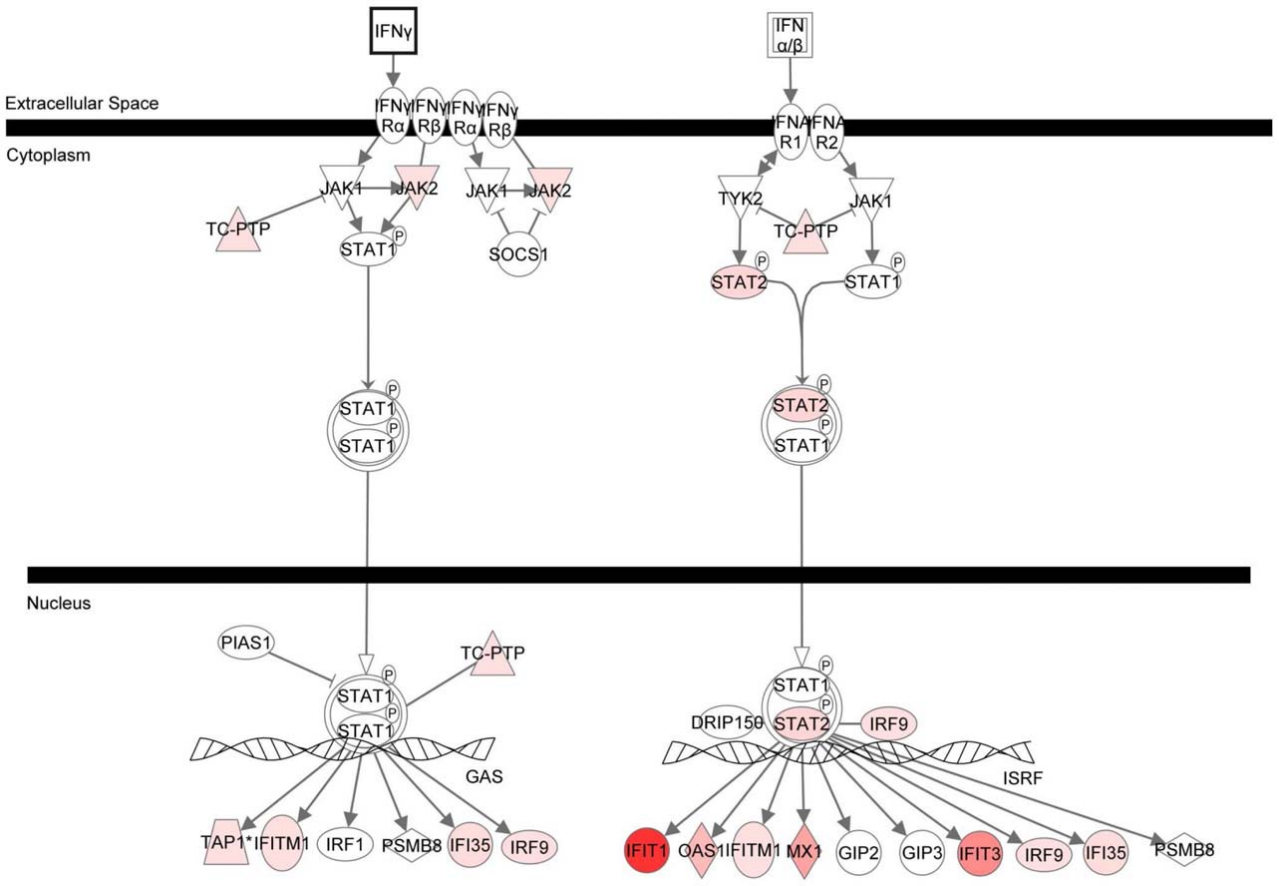
Type I interferon transcripts are induced by parasites on NK cells. Ingenuity Pathways Analysis identified the canonical pathway “Interferon Signaling” as highly associated with the 3D7-regulated genes on NK cells. Mainly IFN-αβ transcripts are induced. The differentially-regulated genes are marked in red (up-regulation) or green (down-regulation).

Ingenuity Systems generated top networks, with a score higher than 40, based on the analysis of the iRBCs-regulated NK genes. *Antimicrobial/Inflammatory Responses and Infection Mechanism* are the main functions associated with the top-scoring networks (Table 2 and Table S2). Moreover, *Interferon signaling* (p = 2,17E-14) and *Activation of IRF by cytosolic pattern recognition receptors* (p = 2,9E-09) were identified as the top canonical pathways linked to the modulated genes (Figure 2A and Table S3). The highest strength of association was found with the *Interferon Signaling* canonical pathway. Eleven of the 30 molecules that compose the pathway were regulated on NK cells by co-culture with parasites (ratio: 0.367). The pathway and the modulated genes are depicted in Figure 3.

**Table 2:** Top networks and functions associated with 3D7- or IL-12/IL-18-induced transcripts on NK cells.

NW ID	Network functions related to 3D7-induced transcripts	Score
1	Antimicrobial Response, Inflammatory Response, Infection Mechanism	61
2	Antimicrobial Response, Inflammatory Response, Infection Mechanism	54
3	Infection Mechanism, Organismal Injury and Abnormalities, RNA Damage and Repair	44
4	Infection Mechanism, Antimicrobial Response, Inflammatory Response	40
5	Post-Translational Modification, Protein Folding, Cell Morphology	31

NW ID	Network functions related to IL-12/IL-18-induced transcripts	Score
1	Gene Expression, Infection Mechanism, RNA Post-Transcriptional Modification	50
2	Cell Death, Post-Translational Modification, Protein Folding	43
3	DNA Replication, Recombination, and Repair, Cellular Growth and Proliferation	40
4	Cell Death, Genetic Disorder, Immunological Disease	37
5	Cell Morphology, Hematological System Development and Function, Cancer	36

NW ID-network identification; IL-interleukin.

### Type II interferon-induced NK genes are up-regulated by IL-12 and IL-18

To compare patterns of NK cell activation, PBMCs were also treated with IL-12 and IL-18, well-described NK cell stimulators. Treatment with the cytokine mix resulted in the regulation of 576 NK genes in both donors E and K. Down-regulated genes totalled 160, whereas 416 genes were up-regulated (Table S4). Modulated genes included those related to the immune response (*IFN-γ*, *CD25*), signal transduction (*P2RX5*, *MT2A*, *KLRF1*), complement activation (*CD55*), antigen presentation (*CD83*), chemotaxis (*CCR4*, *CXCL10*, *CX3CR1*), DNA replication/repair (*CHEK1*, *TYMS*), transcription (*IRF8*, *MYC*), and cytokines (*IL26*, *IL6*), among others. The most up and down-regulated genes share similar molecular as well as biological functions such as receptor activity and signal transduction/immune response, respectively (Table 3; fold change ≥12 and fold change ≤−5.5).

**Table 3:** Top up/down-regulated genes on NK cells due to treatment with IL-12 and IL-18.

Symbol	Aff. ID	FC	Location	Type(s)
IFNG	7964787	92,46	Extr. Space	cytokine
IL2RA	7931914	37,33	Plasma MB	Tmb R
MIR155HG	8068022	34,30	unknown	other
SLC27A2	7983650	20,71	Cytoplasm	transporter
DPP4	8056222	17,57	Plasma MB	peptidase
CD274	8154233	16,19	Plasma MB	other
CDC6	8007071	14,25	Nucleus	other
MYO1B	8047127	13,25	Cytoplasm	other
P2RX5	8011415	13,10	Plasma MB	ion channel
TNFSF4	7922343	13,05	Extr. Space	cytokine
PTGDR	7974363	−5,54	Plasma MB	GPCR
YPEL1	8074780	−5,56	Nucleus	enzyme
FGFBP2	8099471	−5,63	Extr. Space	other
PIK3IP1	8075483	−5,78	unknown	other
KLHL24	8084219	−5,79	unknown	other
AHNAK	7948667	−5,96	Nucleus	other
CX3CR1	8086344	−6,68	Plasma MB	GPCR
FAIM3	7923917	−7,37	unknown	other
SH2D1B	7921900	−7,56	unknown	other
KLRF1	7953892	−10,20	Plasma MB	Tmb R
IL7R	8104901	−11,12	Plasma MB	Tmb R

IL-interleukin; Aff. ID-affymetrix identification; FC-fold change; MB-membrane; Extr.-extracellular; Tmb R-transmembrane receptor; GPCR-G protein coupled receptor.

### Top Networks and Pathways related to IL-12 and IL-18-induced genes

The highest score upon analysis of the IL-12 and IL-18 modulated genes was given to the network that listed *Gene Expression*, *Infection Mechanism*, *RNA Post-Transcriptional Modification* as associated functions (score: 50; Table 2 and Table S5). The most significant canonical pathways obtained from Ingenuity analysis were *TREM1 Signaling* (p = 2,69E-07) and the *Protein Ubiquitination Pathway* (p = 3,38E-07; Table S6). The former canonical pathway is composed of 69 molecules and 13 out of those were found to be modulated in NK cells, resulting in a high strength of association (ratio: 0,188; Figure 2B). The top canonical pathway and its modulated genes are depicted in Figure S2.

### Gene expression similarities between iRBCs- and IL mix-treated NK cells

There were about 400 additional transcripts regulated by IL-12 and IL-18 in comparison to iRBCs-induced genes. In total, only 40 were modulated by both treatments. Among those are transmembrane receptors (*IL2RA*, *CCR1*, *IL12RB2*), cytokines (*CXCL10*, *CCL3*), transcription regulators (*STAT3*, *LBA1*) and enzymes (*PTPN2*, *HSPA8*).

**Figure 4:**
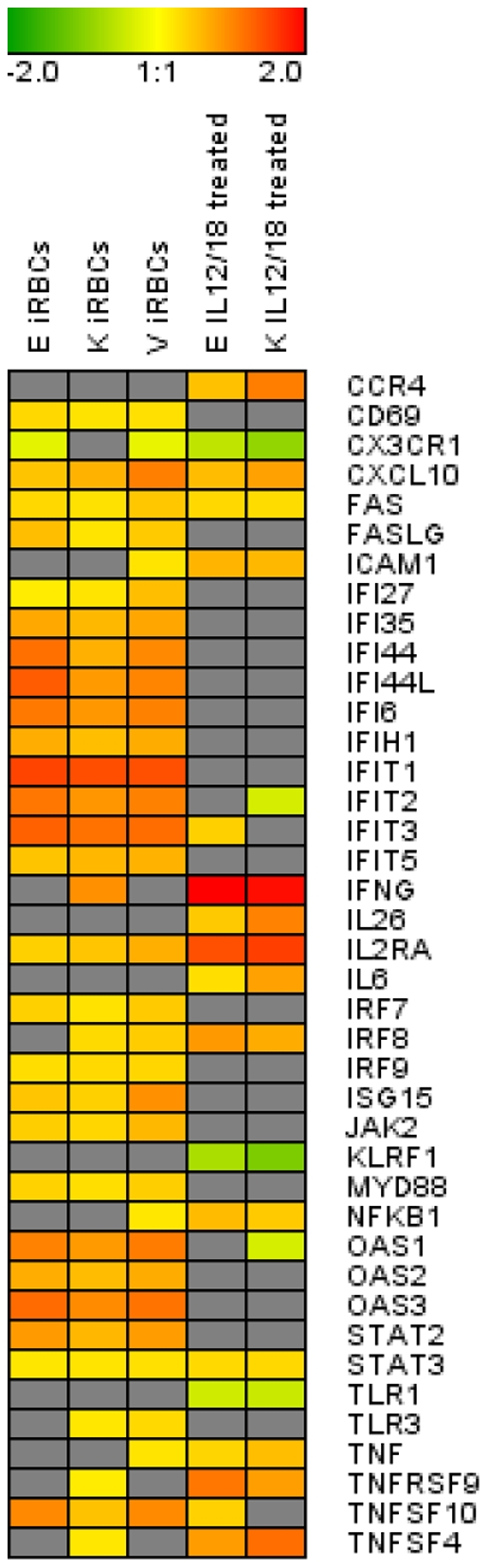
Comparison of the gene expression patterns between donors and treatments. Members of the *Interferon signaling pathway*, the *TREM-1 signaling pathway*, and other genes that play a role in the immune response were selected. The image depicts the log of the fold change of NK genes regulated due to co-culture with 3D7-infected erythrocytes (iRBCs) or with the cytokines IL-12 andIL-18. The expression profile is shown for the three different donors (E, K, and V).

For a general overview, members of the *Interferon signaling pathway*, *TREM-1 signaling pathway* and other genes that play a role in immune response were arranged in a heatmap (Figure 4). The image depicts the comparison of the gene fold change between the three different donors in response to the treatment type. It is clear that, although the same pattern of gene regulation is generally maintained among the donors within the different treatments, parasites and IL-12/IL-18 affect the transcription of NK genes in a different manner.

### Influence of uninfected erythrocytes on NK cells

Since the RBCs and the NK cells used in this study are from different donors, PBMCs from donor E and K were incubated with uRBCs in order to control the allogeneic responses that might affect gene expression in NK cells. This analysis showed that in total only nine genes were modulated due to uRBCs treatment. NK cells from donor E up-regulated six genes, whereas donor K cells showed up-regulation of three different genes. The biological processes or molecular functions of some of these genes have not been described (*RNU5E*, *SNORD47*), and others are known to play a role in RNA splicing (*SNRPN*) and translation (*EEF1A1*).

### Patterns of NK activation and inhibition of parasite growth by expanded NK cells

**Figure 5:**
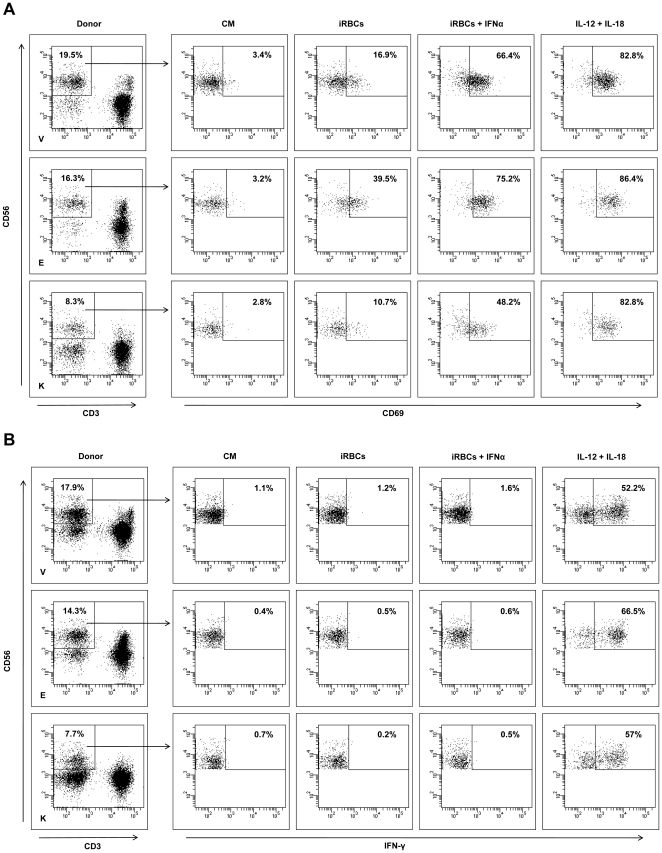
Regulation of CD69 and IFN-γ on NK cells. PBMCs from the three donors were incubated with 3D7 schizont-infected erythrocytes (iRBCs), with iRBCs plus human IFN-α or with a mixture of IL-12 and IL-18, or were leftuntreated in culture medium (CM) for 24 hours and analyzed by flow cytometry. The gating strategy for NK cells (CD56^+^CD3^−^ lymphocytes) and the percentages of CD69^+^ (**A**) and IFN-γ^+^ (**B**) cells for each treatment are depicted. Upper rows: donor V; middle rows: donor E; lower rows: donor K.

The activation characteristics of NK cells from the three donors studied were next examined. NK cell up-regulation of the CD69 membrane surface protein and production of IFN-γ were examined in response to incubation with iRBCs, iRBCs plus IFN-α, IL-12 and IL-18, and culture medium alone. All the donors' NK cells up-regulated CD69 due to iRBCs incubation, although the strength of the responses differed among donors (Figure 5A). In response to parasite stimulation, only 10.7% of the NK cells from donor K up-regulated CD69, while 16.9% and 39.5%, respectively, of the NK cells from donors V and E responded. The addition of IFN-α to the system contributed to NK activation by increasing the percentage of cells that up-regulated CD69 for all donors. IL-12/IL-18 treatment induced around 80% of the donors' cells to express CD69. None of the co-culture conditions induced IFN-γ release by NK cells to a large extent, except for the IL-12/IL-18 treatment (Figure 5B).

**Figure 6:**
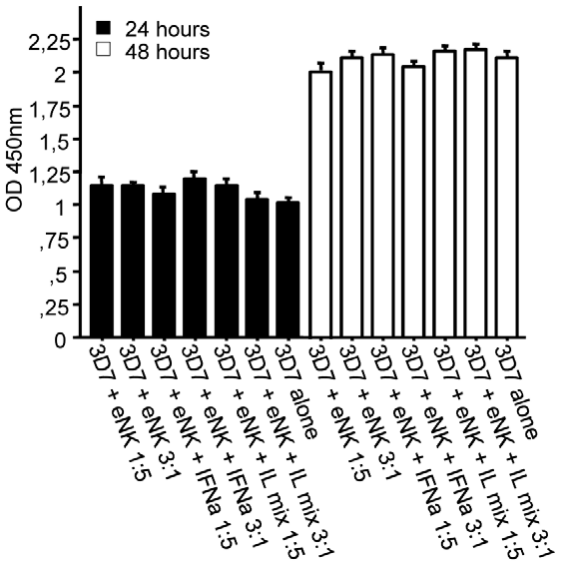
The influence of expanded NK cells and IFN-α on parasite growth. Expanded NK cells (eNK) from donor E were co-cultured with 3D7 ring-infected erythrocytes (iRBCs), with iRBCs plus human IFN-α, or with a mixture of IL-12 and IL-18. Parasites were incubated alone as a control. After 24 h and 48 h, culture samples were frozen at −20°C, thawed and inhibition of parasite growth was tested by HRP2 ELISA assay. Values represent the mean of three different experiments. Ratios are depicted as 3D7∶eNK (1∶3 and 5∶1).

To further examine the cytotoxic characteristics of the cells used in the study, we co-cultured expanded NK cells (eNK) from donor E with 3D7-iRBCs. Neither the 24 h/48 h co-culture time nor the different 3D7∶eNK ratios (1∶5; 3∶1) appeared to have a significant effect on parasitemia. Additionally, parasite growth was not affected by the addition of IFN-α and IL-12/IL-18 to the system (Figure 6).

## Discussion

The objective of this study was to observe the transcriptional changes that malaria parasites impose on NK cells in order to gain a deeper knowledge of the mechanisms behind such interaction. An *in vitro* approach was used to investigate the specific immune response to malaria. Such an approach is widely used in research especially in diseases where primary material is difficult to access. The gene expression profile and activation characteristics of NK cells incubated with iRBCs at a 1∶3 ratio (PBMCs∶iRBCs) were examined at one time point (24 h) after co-culture. These conditions were chosen based on prior observations showing that optimal NK cell IFN-γ production occurs at either 1×10^6^ or 1×10^7^ iRBCs per 10^6^ PBMCs and that the peak of IFN-γ release occurs between 15 and 24 hours after stimulation [3].

First, these results demonstrate that all donors' NK cells have a very similar pattern of gene regulation for each different treatment. An interferon signaling gene expression signature is induced by iRBCs on NK cells, in which genes involved in a pro-inflammatory response, mainly mediated by type I interferon were modulated. A recent microarray study has described the up-regulation of *IFIT1*, *IFIT3*, *and CXCL10* (after 1 h of activation) and *IFI44*, *IFIT2*, *and ISG20* (after 18 h of activation) in IFN-α-treated whole PBMCs from healthy donors. Arrays performed with isolated cell subsets (NK cells, monocytes and T cells) showed the up-regulation of *OAS2*, *OASL*, *ISG20* and *IFI44* [14]. Another group has reported the up-regulation of *TNFSF10* (TRAIL), *IFIT* and *OAS* genes in NK cells isolated from IFN-α-2b-stimulated PBMCs from five healthy donors [15]. The expression profile of IFN-α-treated cells in these studies was very similar to the profile detected in iRBCs-activated NK cells in the current study, which did not utilize IFN-α. Therefore, such similarities provide support of a clear iRBCs-induced type I interferon-related response in NK cells. Moreover, the regulated molecules were linked to top canonical pathways, all belonging to the “cellular immune response” category. Some of the regulated genes were not yet assigned to a clear role in this category, but most genes were linked to well-known biological functions, mainly in infection control and inflammation. As in systemic lupus erythematosus (LE) [16], [17], such inflammatory components were recently detected as increased IFN-α/β-inducible genes in the blood of patients with Tuberculosis (TB), especially in their purified neutrophils [18]. Its correlation with disease severity provided primary data supporting a role for type I IFN in the pathogenesis of human disease. Here, we have observed that mostly the same transcripts found in the mentioned studies were overexpressed in our NK cells due to parasite co-incubation. It is difficult to extrapolate the LE/TB findings to malaria without confirmation with longitudinal studies; however, it is possible that the role of type I interferon signaling in diseases caused by intracellular pathogens will be a marker of disease progression and immune response development.

In this study, the IFIT family was found to be among the most highly up-regulated NK genes induced by parasites. To date, little is known about their function. Most of the evidence characterizes these proteins as inhibitors of cellular and viral processes such as protein translation [19]. Recent findings indicate that IFIT proteins are substantially induced during infection possibly reflecting a functional role. A complex formed by IFIT1, 2 and 3 was observed to exert antiviral activity by physically engaging microbial triphosphorylated-RNA suggesting that these proteins possibly have the ability to bind to various types of nucleic acids of other diverse microbes [20]. *Plasmodium* DNA, therefore, could be one target. In fact, DNA sensing and its relation to type I interferons have recently been revealed to be important in innate immunity to malaria [21]. *Plasmodium* genomic DNA, rich in AT motifs, was shown to generate type I interferon through two suggested innate pathways (a TLR9- and a STING- driven) which converge on the IRFs to regulate IFN gene transcription. Interferon type I, in turn, could possibly influence the outcome of the disease.

Instead of IFN-α/β-related genes though, we expected to observe a direct up-regulation of the *IFN-γ* gene in all donors treated with iRBCs (as was detected with the IL-12/IL-18 treated NK cells). To our surprise, NK cells from only one donor (donor K) up-regulated the *IFN-γ* gene, and the fold change was much lower than that induced by IL-12 and IL-18. Nevertheless, it is very likely that this 3D7-mediated induction of type I-related genes reflects the first steps of a cascade of events leading to IFN-γ release. In the case of a viral infection, there is consensus that the activation of NK cells is critically dependent on type I IFN signaling *in vivo* and that this activation is achieved by its direct action on NK cells [22]. Others have shown that type I IFNs are an early and critical regulator of NK cell number, activation and antitumor activity and that, in combination with IL-18, type I IFN plays an important role in inducing IFN-γ production by NK cells [23], [24]. In addition to overlapping with type II interferon at multiple levels of the JAK/STAT signaling pathway, type I interferons have unique regulatory mechanisms for both their own signaling as well as IFN-γ signaling [25]. A very recent report showing the responses of human PBMCs to stimulation with type I and II interferons, among other cytokines, is in agreement with this co-induction concept. The authors describe the responses to IFN-γ as being restricted to a subset of type I interferon-inducible genes whereas responses to type I interferon were highly stereotyped and resulted in the up-regulation of genes such as *OAS1-3*, *MX1/2*, *CXCL10*, *STAT1/2*, *IRF2/7* and *IFIT1-5*. However, after four hours of IFN-α treatment, transcripts of type II interferon itself were induced, which the authors suggested might play a role in the initiation of an IFN-γ-dependent transcriptional programs in type I IFN-treated cells [26]. Along these lines, *L. major*-induced IFN-α/β was suggested to mediate key events of the innate response to the parasite. NK cell cytotoxicity and IFN-γ secretion early in infection were shown to be decreased in the event of type I interferon blockage in mice [27]. In a recent study with *L. mexicana* infection, type I interferon was also described to promote the early IFN-γ and IL10 expression [28].

*P. falciparum*-mediated IFN-α responses have been previously reported by few *in vivo* and *in vitro* studies [29]–[31]. In accordance with our findings, new microarray evidence show that expression profiling of PBMCs derived from patients with *P. falciparum* malaria show elevated expression of interferon-inducible genes (ISGs) [21]. The study further confirms that PBMCs stimulated with iRBCs induce IFN-α at the protein level and IFN-β mRNA, suggesting a possible role for type I interferons in malaria. Although their gene expression profiling was performed with a mixed group of cells and hence cannot be traced to one specific cell population, the NK cells in the present study appear to respond in concert with PBMCs from malaria-infected individuals in that previous study. Furthermore, human plasmatocytoid dendritic cells (pDCs) were suggested to produce IFN-α in response to *P. falciparum-*schizonts, which in turn promote γδ T cell proliferation and IFN-γ production [32]. Microarray analysis of whole-blood cells from *P. chabaudi*-infected mice [33] demonstrated that the predominant responses at about 16 h to 24 h post-infection were dominated by interferon-induced genes and that after 32 hours there was a dramatic change in the regulated transcripts. However, there are contradictory studies regarding the importance of IFN-α in the immune response against malaria. A recent study has described that neither pDCs nor IFN-α/β were essential for parasite clearance as mice depleted of pDCs or IFN-α/β receptor knock-out mice could control *P. chabaudi* infection [34]. In contrast, experimental evidence suggested that IFN-α treatment of *P. berghei*-infected mice has a protective effect on the evolution of cerebral malaria and inhibits the development of *P. yoelli* blood-stage infections in mice [35], [36]. In addition, polymorphisms in the IFN-α receptor 1 were associated with protection against cerebral malaria in humans [37]. High titers of antiviral activity have been reported to be due to IFN-α, and a positive correlation between degree of parasitemia, interferon titers and NK cell activity was observed in acutely ill *P. falciparum*-infected children [29]. IFN-α, in combination with iRBCs, boosted the up-regulation of CD69 on NK cells but did not up-regulate IFN-γ in the present study. Additionally, when testing the cytotoxicity of expanded NK cells from Donor E against *Plasmodium*, no significant interference in parasite growth was observed, even with the addition of IFN-α. Such lack of cytotoxicity was likely due to donor-related characteristics (low IFN-γ responder) but it will be important to determine the reason that expanded NK cells treated with IL-12/IL-18 (which lead to IFN-γ release) did not inhibit parasite growth. As mentioned before, others have described that the peak of IFN-γ induction occurs around 15–24 h after co-culture with parasites and that this response is dependent on cross-talk with other cells. Thus, it would be worth observing whether HPR2 is suppressed at earlier time points than 24 h and 48 h after co-culture and whether the addition of accessory cells to the system would interfere with parasite growth. To further investigate the importance of IFN-α on the NK response against parasites, 3D7-iRBCs were co-cultured with NK92 (NK cell line) in which *IRF9*, *STAT1* or *STAT2* were knocked down by siRNA and RT-PCR was used to verify the suppression of EBA-175 and BAEBL/EBA-140, which are vital parasite genes involved in invasion (our unpublished observations). However, no differences were observed in cytotoxicity of the siRNA -transfected cells against 3D7. One potential explanation for this is the fact that, due to the difficulties in obtaining large amounts of fresh NK cells, these experiments were performed with an NK cell line, which might not reflect physiological conditions. Another reason could be the choice to evaluate cytotoxicity by the NK population, although there is still considerable debate regarding the importance of NK and T cells in immunity to malaria. A very recent study with *P. chaubadi*-infected mice shows that the suppression of infection is dependent on γδ T cells and independent of NK cells [38]. Conversely, a study with a large cohort of malaria-naive donors shows that the majority of IFN-γ^+^ T cells are αβ and not γδ T cells. Moreover, the authors of that study observed that NK cells dominate the early IFN-γ response (around 18 h), that NK and T cells contribute equally to the response at 24 h, and that T cells dominate from there-after [39].

The combination of IL-12 and IL-18 augments NK cell activity and stimulates NK production of IFN-γ, a cytokine suggested to control *P. falciparum* infection [3]–[5]. PBMCs stimulation with high doses of IL-12 and IL-18 (as performed in this study) was previously shown to up-regulate NK cell expression of CD69 and CD25, and to stimulate the release of IFN-γ [5]. We were interested in determining whether there were similarities between the transcripts induced by IL-12/IL-18 and iRBCs. However, this was not the case. IL-12/IL-18 treatment induced genes strongly correlated to the signaling pathway triggered by *TREM 1*, an immune regulatory molecule that plays a role in innate and adaptive immune response [40]. The molecule is expressed on monocytes/macrophages, dendritic cells, NK cells, and neutrophils [41], [42] and its activation triggers molecules involved in cell-to-cell signaling/interactions and inflammatory responses (including CD83, IL-6 and TNF among others). NK cells induced by iRBCs in our study also modulated some genes related to this pathway although not as strongly as the IL-12/IL-18 treatment. The second strongest gene association was found with the protein ubiquitination pathway, which consists of a concerted action of enzymes indispensable for the rapid removal of proteins, the regulation of gene transcription, translational quality control and immune surveillance, to mention some of the functions. A prominent molecule in immune surveillance is IFN-γ, which was found to be the top molecule (FC = 92,460) up-regulated by IL-12/IL-18 treatment in this study. The ubiquitin-proteasome system is essential for antigen presentation on MHC class I molecules and this process is enhanced by IFN-γ. This cytokine induces immune cells to express immunoproteasomes that impose changes on the normal cascade of actions of the pathway, consequently leading to the stimulation of host defence [43].

Overall, this study provides evidence that *P. falciparum* parasites induce IFN-α-associated transcripts in human NK cells within the first 24 hours of interaction. This study also demonstrated that the NK transcriptional changes induced by IL-12 and IL-18 are diverse from those induced by 3D7. Whether both patterns of expression converge at one stage and whether IFN-α-related transcripts result in IFN-γ signaling, should be further investigated by microarrays and functional studies at different time points. The role of IFN-α in malaria is still controversial and understudied. This study suggests inherent regulatory molecules of the NK response to parasites that might be potential targets to be considered in malaria vaccine development.

## Supporting Information

Figure S1 **Purity of the isolated NK cells measured by FACS.** Values represent the percentage of pure NK cells (CD56^+^CD3^−^) before and after isolation within the four different co-culture conditions: CM: culture medium only; iRBCs: +infected erythrocytes; uRBCs: +uninfected erythrocytes; IL-12+IL-18: IL-12 and IL-18. E1, E2 and E3 represent the three replicates for donor E; K1, K2 and K3 represent the three replicates for donor K and V1, V2 and V3 represent the three replicates for donor V.
(TIF)

Figure S2 **IL-12/IL-18 treatment of NK cells induces transcripts related to the TREM-1 signaling pathway.** The “Triggering receptor expressed in myeloid cell 1” (TREM-1) signaling pathway was identified by the Ingenuity Pathways knowledge base as highly associated with the IL-12/IL-18-regulated genes on NK cells. Up-regulated genes are highlighted in red and the down-regulated genes are highlighted in green.
(TIF)

Table S1 **Complete list of** ***P. falciparum*** **-iRBCs induced genes.**
(XLS)

Table S2 **Complete list of Networks related to** ***P. falciparum*** **-iRBCs induced genes.**
(XLS)

Table S3 **Complete list of Canonical Pathways related to** ***P. falciparum*** **-iRBCs induced genes.**
(XLS)

Table S4 **Complete list of IL-12/IL-18-induced genes.**
(XLS)

Table S5 **Complete list of Networks related to IL-12/IL-18-induced genes.**
(XLS)

Table S6 **Complete list of Canonical Pathways related to IL-12/IL-18-induced genes.**
(XLS)

## References

[1] Stevenson MM, Riley EM (2004). Innate immunity to malaria.. Nat Rev Immunol.

[2] Urban BC, Ing R, Stevenson MM (2005). Early interactions between blood-stage plasmodium parasites and the immune system.. Curr Top Microbiol Immunol.

[3] Artavanis-Tsakonas K, Riley EM (2002). Innate immune response to malaria: rapid induction of IFN-gamma from human NK cells by live Plasmodium falciparum-infected erythrocytes.. J Immunol.

[4] Artavanis-Tsakonas K, Eleme K, McQueen KL, Cheng NW, Parham P (2003). Activation of a subset of human NK cells upon contact with Plasmodium falciparum-infected erythrocytes.. J Immunol.

[5] Korbel DS, Newman KC, Almeida CR, Davis DM, Riley EM (2005). Heterogeneous human NK cell responses to Plasmodium falciparum-infected erythrocytes.. J Immunol.

[6] Orago AS, Facer CA (1991). Cytotoxicity of human natural killer (NK) cell subsets for Plasmodium falciparum erythrocytic schizonts: stimulation by cytokines and inhibition by neomycin.. Clin Exp Immunol.

[7] Mavoungou E, Luty AJ, Kremsner PG (2003). Natural killer (NK) cell-mediated cytolysis of Plasmodium falciparum-infected human red blood cells in vitro.. Eur Cytokine Netw.

[8] Mavoungou E, Held J, Mewono L, Kremsner PG (2007). A Duffy binding-like domain is involved in the NKp30-mediated recognition of Plasmodium falciparum-parasitized erythrocytes by natural killer cells.. J Infect Dis.

[9] Baratin M, Roetynck S, Lepolard C, Falk C, Sawadogo S (2005). Natural killer cell and macrophage cooperation in MyD88-dependent innate responses to Plasmodium falciparum.. Proc Natl Acad Sci U S A.

[10] Baratin M, Roetynck S, Pouvelle B, Lemmers C, Viebig NK (2007). Dissection of the role of PfEMP1 and ICAM-1 in the sensing of plasmodium falciparum-infected erythrocytes by natural killer cells.. PLoS ONE.

[11] Newman KC, Korbel DS, Hafalla JC, Riley EM (2006). Cross-talk with myeloid accessory cells regulates human natural killer cell interferon-gamma responses to malaria.. PLoS Pathog.

[12] Trager W, Jensen JB (2005). Human malaria parasites in continuous culture. 1976.. J Parasitol.

[13] Noedl H, Bronnert J, Yingyuen K, Attlmayr B, Kollaritsch H (2005). Simple histidine-rich protein 2 double-site sandwich enzyme-linked immunosorbent assay for use in malaria drug sensitivity testing.. Antimicrob Agents Chemother.

[14] Zimmerer JM, Lesinski GB, Ruppert AS, Radmacher MD, Noble C (2008). Gene expression profiling reveals similarities between the in vitro and in vivo responses of immune effector cells to IFN-alpha.. Clin Cancer Res.

[15] Stegmann KA, Bjorkstrom NK, Veber H, Ciesek S, Riese P (2010). Interferon-alpha-induced TRAIL on natural killer cells is associated with control of hepatitis C virus infection.. Gastroenterology.

[16] Baechler EC, Batliwalla FM, Karypis G, Gaffney PM, Ortmann WA (2003). Interferon-inducible gene expression signature in peripheral blood cells of patients with severe lupus.. Proc Natl Acad Sci U S A.

[17] Petri M, Singh S, Tesfasyone H, Dedrick R, Fry K (2009). Longitudinal expression of type I interferon responsive genes in systemic lupus erythematosus.. Lupus.

[18] Berry MP, Graham CM, McNab FW, Xu Z, Bloch SA (2010). An interferon-inducible neutrophil-driven blood transcriptional signature in human tuberculosis.. Nature.

[19] Fensterl V, Sen GC (2011). The ISG56/IFIT1 gene family.. J Interferon Cytokine Res.

[20] Pichlmair A, Lassnig C, Eberle CA, Gorna MW, Baumann CL (2011). IFIT1 is an antiviral protein that recognizes 5′-triphosphate RNA.. Nat Immunol.

[21] Sharma S, Deoliveira RB, Kalantari P, Parroche P, Goutagny N (2011). Innate Immune Recognition of an AT-Rich Stem-Loop DNA Motif in the Plasmodium falciparum Genome.. Immunity.

[22] Zhu J, Huang X, Yang Y (2008). A critical role for type I IFN-dependent NK cell activation in innate immune elimination of adenoviral vectors in vivo.. Mol Ther.

[23] Swann JB, Hayakawa Y, Zerafa N, Sheehan KC, Scott B (2007). Type I IFN contributes to NK cell homeostasis, activation, and antitumor function.. J Immunol.

[24] Matikainen S, Paananen A, Miettinen M, Kurimoto M, Timonen T (2001). IFN-alpha and IL-18 synergistically enhance IFN-gamma production in human NK cells: differential regulation of Stat4 activation and IFN-gamma gene expression by IFN-alpha and IL-12.. Eur J Immunol.

[25] Theofilopoulos AN, Baccala R, Beutler B, Kono DH (2005). Type I interferons (alpha/beta) in immunity and autoimmunity.. Annu Rev Immunol.

[26] Waddell SJ, Popper SJ, Rubins KH, Griffiths MJ, Brown PO (2010). Dissecting interferon-induced transcriptional programs in human peripheral blood cells.. PLoS ONE.

[27] Diefenbach A, Schindler H, Donhauser N, Lorenz E, Laskay T (1998). Type 1 interferon (IFNalpha/beta) and type 2 nitric oxide synthase regulate the innate immune response to a protozoan parasite.. Immunity.

[28] Buxbaum LU (2010). Type I IFNs promote the early IFN-gamma response and the IL-10 response in Leishmania mexicana infection.. Parasite Immunol.

[29] Ojo-Amaize EA, Salimonu LS, Williams AI, Akinwolere OA, Shabo R (1981). Positive correlation between degree of parasitemia, interferon titers, and natural killer cell activity in Plasmodium falciparum-infected children.. J Immunol.

[30] Ronnblom L, Ojo-Amaize EA, Franzen L, Wigzell H, Alm GV (1983). Plasmodium falciparum parasites induce interferon production in human peripheral blood ‘null’ cells in vitro.. Parasite Immunol.

[31] Luty AJ, Perkins DJ, Lell B, Schmidt-Ott R, Lehman LG (2000). Low interleukin-12 activity in severe Plasmodium falciparum malaria.. Infect Immun.

[32] Pichyangkul S, Yongvanitchit K, Kum-arb U, Hemmi H, Akira S (2004). Malaria blood stage parasites activate human plasmacytoid dendritic cells and murine dendritic cells through a Toll-like receptor 9-dependent pathway.. J Immunol.

[33] Kim CC, Parikh S, Sun JC, Myrick A, Lanier LL (2008). Experimental malaria infection triggers early expansion of natural killer cells.. Infect Immun.

[34] Voisine C, Mastelic B, Sponaas AM, Langhorne J (2010). Classical CD11c+ dendritic cells, not plasmacytoid dendritic cells, induce T cell responses to Plasmodium chabaudi malaria.. Int J Parasitol.

[35] Vigario AM, Belnoue E, Cumano A, Marussig M, Miltgen F (2001). Inhibition of Plasmodium yoelii blood-stage malaria by interferon alpha through the inhibition of the production of its target cell, the reticulocyte.. Blood.

[36] Vigario AM, Belnoue E, Gruner AC, Mauduit M, Kayibanda M (2007). Recombinant human IFN-alpha inhibits cerebral malaria and reduces parasite burden in mice.. J Immunol.

[37] Aucan C, Walley AJ, Hennig BJ, Fitness J, Frodsham A (2003). Interferon-alpha receptor-1 (IFNAR1) variants are associated with protection against cerebral malaria in the Gambia.. Genes Immun.

[38] Weidanz WP, LaFleur G, Brown A, Burns JM, Gramaglia I (2010). Gammadelta T cells but not NK cells are essential for cell-mediated immunity against Plasmodium chabaudi malaria.. Infect Immun.

[39] Horowitz A, Newman KC, Evans JH, Korbel DS, Davis DM (2010). Cross-talk between T cells and NK cells generates rapid effector responses to Plasmodium falciparum-infected erythrocytes.. J Immunol.

[40] Bleharski JR, Kiessler V, Buonsanti C, Sieling PA, Stenger S (2003). A role for triggering receptor expressed on myeloid cells-1 in host defense during the early-induced and adaptive phases of the immune response.. J Immunol.

[41] Bouchon A, Dietrich J, Colonna M (2000). Cutting edge: inflammatory responses can be triggered by TREM-1, a novel receptor expressed on neutrophils and monocytes.. J Immunol.

[42] Cantoni C, Bottino C, Vitale M, Pessino A, Augugliaro R (1999). NKp44, a triggering receptor involved in tumor cell lysis by activated human natural killer cells, is a novel member of the immunoglobulin superfamily.. J Exp Med.

[43] Lecker SH, Goldberg AL, Mitch WE (2006). Protein degradation by the ubiquitin-proteasome pathway in normal and disease states.. J Am Soc Nephrol.

