# Supplementary figures and images for: Plasmodium falciparum-Infected Erythrocytes and IL-12/IL-18 Induce Diverse Transcriptomes in Human NK Cells: IFN-α/β Pathway versus TREM Signaling
Source: PLoS One. 2011 Sep 16;6(9):e24963. doi: 10.1371/journal.pone.0024963 (PMC3174986; doi:10.1371/journal.pone.0024963)

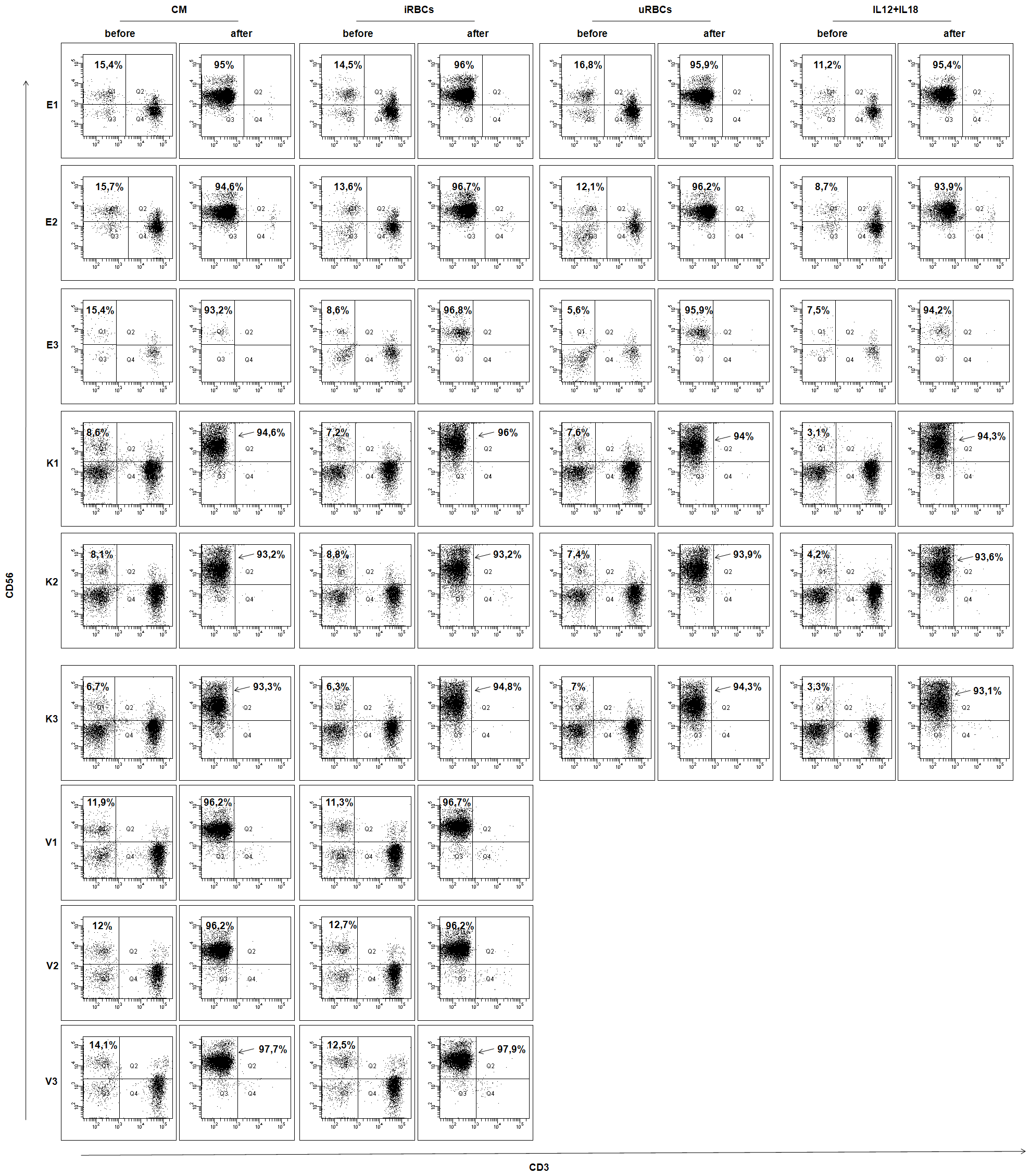

Supplement: Figure S1 — Purity of the isolated NK cells measured by FACS. Values represent the percentage of pure NK cells (CD56+CD3−) before and after isolation within the four different co-culture conditions: CM: culture medium only; iRBCs: +infected erythrocytes; uRBCs: +uninfected erythrocytes; IL-12+IL-18: IL-12 and IL-18. E1, E2 and E3 represent the three replicates for donor E; K1, K2 and K3 represent the three replicates for donor K and V1, V2 and V3 represent the three replicates for donor V. (TIF) [file pone.0024963.s001.tif]

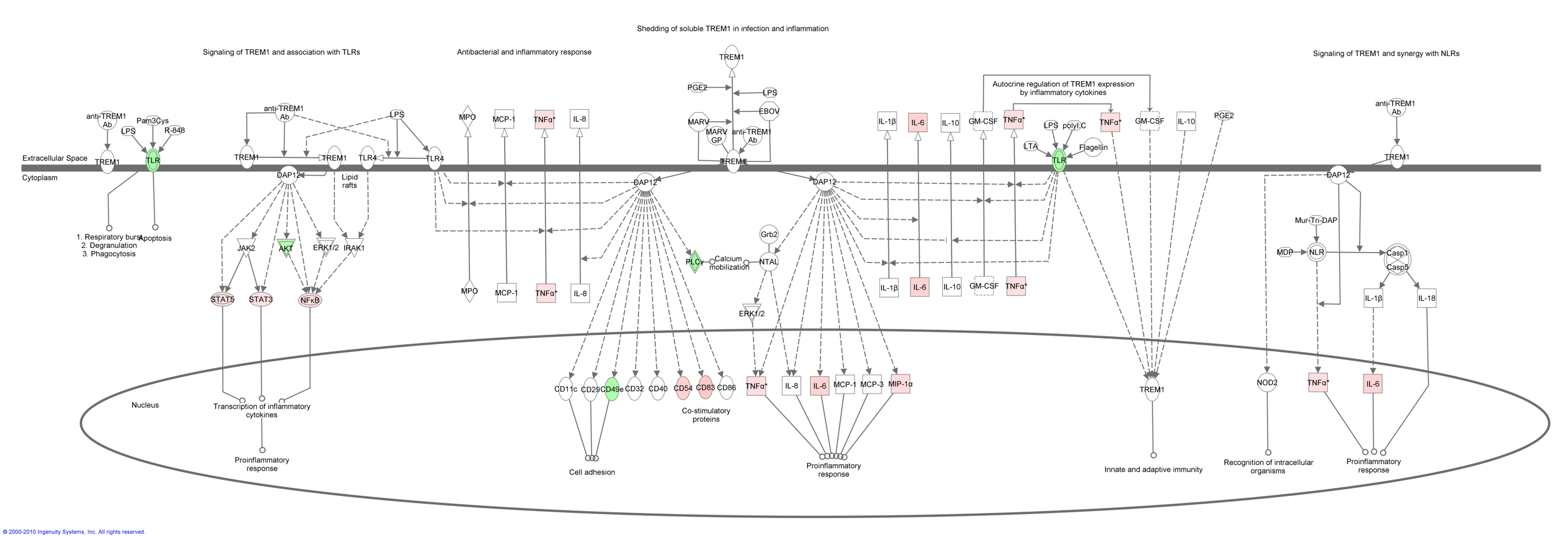

Supplement: Figure S2 — IL-12/IL-18 treatment of NK cells induces transcripts related to the TREM-1 signaling pathway. The “Triggering receptor expressed in myeloid cell 1” (TREM-1) signaling pathway was identified by the Ingenuity Pathways knowledge base as highly associated with the IL-12/IL-18-regulated genes on NK cells. Up-regulated genes are highlighted in red and the down-regulated genes are highlighted in green. (TIF) [file pone.0024963.s002.tif]
